# Predictive factors of melanoma thickness^☆^

**DOI:** 10.1016/j.abd.2021.12.002

**Published:** 2022-06-25

**Authors:** Ana Rita Carreiro Silva, Ricardo José David Costa Vieira

**Affiliations:** a Faculty of Medicine, Universidade de Coimbra, Coimbra, Portugal; b Dermatological Surgery Unit, Centro Hospitalar e Universitário de Coimbra, Coimbra, Portugal

**Keywords:** Dermoscopy, Melanoma, Sentinel lymph node biopsy

## Abstract

**Background:**

Melanoma thickness is a relevant prognostic marker that is crucial for staging and its calculation relies on the histopathological examination. There is a risk of thickness underestimation with an incisional biopsy if the latter is not performed on a tumor area where the thickness is maximal. This occurrence may have an impact on a therapeutic decision, particularly regarding the excision margins and the need for sentinel lymph node biopsy.

**Objective:**

To assess the association between melanoma thickness and dermoscopic, demographic, epidemiological and clinical variables, aiming to identify predictive factors of thickness >1 mm.

**Methods:**

This was an observational and cross-sectional study, carried out on patients diagnosed with melanoma, from a single center over a time span of four years. Anatomopathological (thickness), dermoscopic, demographic, epidemiological, and clinical variables were collected. The associations between the variables with melanoma thickness were assessed.

**Results:**

A total of 119 patients were included. The presence of atypical vessels on the dermoscopic examination was an independent predictive factor of thickness >1 mm. Conversely, an atypical reticular pattern predicted melanoma thickness <1 mm. The presence of ephelides and a previous history of sunburn were also associated with melanomas thinner than 1 mm in the univariate analysis.

**Study limitations:**

The lack of data related to some variables and the absence of an optimal correlation between the dermoscopic and the anatomopathological examination constituted study limitations.

**Conclusion:**

An atypical vascular pattern on dermoscopy is associated with thickness >1 mm, helping with the choice of the optimal site to perform an incisional biopsy when an excisional biopsy is not feasible.

## Introduction

One of the main prognostic factors for melanoma is the thickness of the primary tumor, measured in millimeters from the granular layer of the epidermis or base of a superficial ulceration to the deepest location of the malignant cells, excluding follicular invasion and with an approximation of 0.1 mm.^1^, ^2^ The greater the thickness, the greater the risk of metastasis.^3^ For a thickness <1.0 mm, in the absence of ulceration and a number of mitoses >1 per mm^2^, the sentinel lymph node positivity rate is less than 6%, being at least 14% for a thickness >1.0 mm. Therefore, in the latter case, the sentinel lymph node biopsy is recommended.^4^ In addition to the decision on sentinel lymph node screening, the thickness also influences the extension of the resection margin.^3^, ^5^ The biopsy of a lesion suspected of melanoma should be excisional, whenever possible, so that the therapeutic decision can be based on precise staging parameters.^2^ Incisional biopsies can underestimate thickness in 5% to 22% of cases,^6^ based on sampling that does not always represent the area of ​​greatest tumor thickness.

Dermoscopy is a technique that allows increased diagnostic accuracy in melanoma.^7^ Some studies have shown its capacity to infer histopathological characteristics, namely thickness, mitotic index, and the presence of ulceration.^8^, ^9^, ^10^, ^11^, ^12^ However, the most recent literature is not abundant in this context and the concrete application of the results has been little explored.

Thus, the primary objective of this study was to assess the relationship between melanoma thickness and dermoscopic variables and, secondly, between tumor thickness and demographic, epidemiological, and clinical variables, seeking to identify predictive factors of melanoma thickness.

## Material and methods

An analytical, observational, and cross-sectional study was carried out on a sample of patients diagnosed with cutaneous melanoma, at the Dermatology and Venereology Service of Centro Hospitalar e Universitário de Coimbra, between January 2014 and December 2017.

The sample selection considered the following inclusion criteria: definitive diagnosis of cutaneous melanoma located on the head and neck, trunk, upper limb, hand, lower limb, or foot and the presence of clinical data related to the dermoscopic and anatomopathological examinations.

The clinical records of the selected patients were consulted to collect demographic (age and sex), epidemiological (phototype, eye color, hair color at 18 years of age, presence of ephelides, presence of solar lentigines, history of sunburn with erythema and blisters, number of melanocytic nevi >2 mm in size, according to the definition of the International Agency for Research on Cancer, presence of atypical nevi according to the ABCDE criteria and presence of actinic keratoses), clinical (melanoma location, asymmetry, border, color, change in dimension in the last 12 months and change in color in the last 12 months), dermoscopic (dermoscopy pattern, presence of atypical pigment network or pseudo-network, presence of negative network, presence of irregular dots or globules, presence of hypopigmentation, presence of striae or pseudopods, presence of blue-gray veil, presence of atypical vessels and presence of white shiny lines) and anatomopathological (Breslow thickness) data.

The statistical analysis was performed using the SPSS® software, version 25, with a significance level set at 0.05. The variables were represented through their relative and absolute frequency. The chi-square test and Fisher’s test were used to establish associations between the collected variables. Univariate and multivariate binary logistic regression allowed establishing the predictive factors of thickness >1 mm.

The study was carried out according to the recommendations of the Declaration of Helsinki of the World Medical Association and was approved by the Ethics Committee of Centro Hospitalar e Universitário de Coimbra.

This study did not receive any specific funding from public, private or non-profit agencies.

## Results

A total of 314 patients with cutaneous malignant melanoma were diagnosed in the aforementioned time period. One hundred and ninety-five cases were excluded due to a lack of recording of the dermoscopic variables being assessed, and the remaining 119 cases were included.

The demographic and clinical characteristics of the individuals included in the study are shown in Table 1. Most patients (94.1%; n = 112) were at least 40 years old at the time of the diagnosis and were male (51.3%; n = 61). Malignant melanomas were located mainly on the trunk (35.3%; n = 42). Similar frequencies were recorded for melanoma thickness.

**Table 1:** Demographic and clinical characterization of the sample.

Variable, % (n)	(n = 119)
Age	
<40 years	5.9 (7)
≥40 years	94.1 (112)
Sex	
Male	48.7 (58)
Female	51.3 (61)
Location	
Trunk	35.3 (42)
Head and neck	27.7 (33)
Lower limb (except foot)	16.0 (19)
Foot	11.8 (14)
Upper limb (except hand)	5.9 (7)
Hand	3.4 (4)
Thickness	
≤1 mm	49.6 (59)
>1 mm	50.4 (60)

The multicomponent dermoscopic pattern (54.2%) was associated with a thickness >1 mm, while the reticular pattern (52.5%) was the most frequent in melanomas with a thickness <1 mm (p < 0.001). The presence of an atypical pigment network/pseudo-network (86.4%; p < 0.001) and negative pigment network (8.6%; p = 0.027) were associated with a thickness ≤1 mm, while the presence of an atypical vascular pattern (65.0%; p < 0.001) and white shiny lines (18.3%; p = 0.003) was more frequent in melanomas with a thickness >1 mm (Table 2).

**Table 2:** Association between thickness and dermoscopic variables in patients with malignant melanoma.

Variables	Thickness, % (n)		p-value
	≤1 mm (n = 59)	>1 mm (n = 60)	
Pattern			**<0.001**
Homogeneous	0.0 (0)	6.8 (4)	
Multicomponent	27.1 (16)	54.2 (32)	
Reticular	52.5 (31)	10.2 (6)	
Parallel ridge	3.4 (2)	5.1 (3)	
Outro	16.9 (10)	23.7 (14)	
Atypical pigment network/pseudo-network	86.4 (51)	51.7 (31)	**<0.001**
Negative pigment network	8.6 (5)	0.0 (0)	**0.027**
Irregular dots and/or globules	40.7 (24)	36.7 (22)	0.653
Depigmentation/hypopigmentation	27.6 (16)	26.7 (16)	0.911
Striae/Pseudopods	22.4 (13)	15.0 (9)	0.301
Blue-gray veil	28.8 (17)	41.7 (25)	0.142
Atypical vascular pattern	18.6 (11)	65.0 (39)	**<0.001**
White shiny lines	1.7 (1)	18.3 (11)	**0.003**

Table 3 compares the distribution of demographic and epidemiological variables among patients with malignant melanoma with different thicknesses. The frequency of individuals over 40 years old (98.3%; p = 0.049) was significantly higher when the thickness was >1 mm and this characteristic was associated with a lower frequency of ephelides (1.7%; p = 0.032) and previous sunburns (42.1%; p = 0.032). Sex, phototype, eye color, hair color, presence of solar lentigines, number of melanocytic nevi, presence of atypical nevi, and actinic keratoses did not differ significantly in relation to melanoma thickness.

**Table 3:** Association between thickness and demographic and epidemiological variables in patients with malignant melanoma.

Variables	Thickness, % (n)		p-value
	≤1 mm (n = 59)	>1 mm (n = 60)	
Sex			0.312
Male	44.1 (26)	53.3 (32)	
Female	55.9 (33)	46.7 (28)	
Age			0.049
<40 years	10.2 (6)	1.7 (1)	
≥40 years	89.8 (53)	98.3 (59)	
Phototype			0.320
I	1.7 (1)	1.7 (1)	
II	16.9 (10)	30.5 (18)	
III	64.4 (38)	57.6 (34)	
IV	16.9 (10)	10.2 (6)	
Eye color			0.724
Green	18.6 (11)	20.3 (12)	
Brown	61.0 (36)	54.2 (32)	
Blue	18.6 (11)	20.3 (12)	
Black	1.7 (1)	5.1 (3)	
Color of hair at 18 years			0.592
Blonde	10.2 (6)	18.6 (11)	
Gray	11.9 (7)	8.5 (5)	
Black	22.0 (13)	20.3 (12)	
Brown	55.9 (33)	52.5 (31)	
Ephelides	13.6 (8)	1.7 (1)	0.032
Solar lentigines	55.9 (33)	52.5 (31)	0.854
Previous sunburn	62.1 (36)	42.1 (24)	0.032
Number of melanocytic nevi			0.447
<20	79.7 (47)	88.1 (52)	
20‒50	13.6 (8)	8.5 (5)	
>50	6.8 (4)	3.4 (2)	
Atypical nevi	13.6 (8)	6.8 (4)	0.223
Atypical keratoses	22.0 (13)	28.8 (17)	0.398

The absence of pigmentation (amelanotic melanoma) was associated with thickness >1 mm (17.5%; p < 0.001). No significant differences were observed regarding melanoma location, asymmetry, border, color change, and recent growth in relation to thickness (Table 4).

**Table 4:** Association between thickness and clinical variables in patients with malignant melanoma.

Variables	Thickness, % (n)		p-value
	≤1 mm (n = 59)	>1 mm (n = 60)	
Location			0.147
Head and neck	28.8 (17)	26.7 (16)	
Trunk	39.0 (23)	31.7 (19)	
Upper limb (except hand)	6.8 (4)	5.0 (3)	
Hand	3.4 (2)	3.3 (2)	
Lower limb (except foot)	18.6 (11)	13.3 (8)	
Foot	3.4 (2)	20.0 (12)	
Asymmetry			0.725
Symmetrical	11.5 (6)	9.4 (5)	
Asymmetrical	88.5 (46)	90.6 (48)	
Border			0.810
Regular	13.0 (7)	14.5 (8)	
Irregular	87.0 (47)	85.5 (47)	
Color			**<0.001**
Non-pigmented	0.0 (0)	17.5 (10)	
Pigmented	100.0 (56)	82.5 (47)	
Recent change in color	58.3 (28)	59.6 (31)	0.896
Recent growth	74.5 (38)	83.9 (47)	0.229

Table 5 shows the results of the univariate and multivariate logistic regression on predictive factors of thickness >1 mm in patients with malignant melanoma. In the univariate analysis, the presence of ephelides, previous sunburns, and atypical pigment network/pseudo-network were predictive of thickness <1 mm, but the presence of an atypical vascular pattern or white shiny lines was related to thickness >1 mm. The multivariate regression model explained 49.7% of the variance associated with the thickness (R^2^ = 0.497) and identified the atypical vascular pattern (relative risk of 6.621; p < 0.001) as an independent predicitve factor of thickness >1 mm. Instead, atypical pigment network/pseudo-network (relative risk 0.168; p = 0.001) was identified as a predicitve factor of thickness <1 mm.

**Table 5:** Predictive factors of thickness >1 mm in patients with malignant melanoma.

Dimension	Univariate analysis		Multivariate analysis	
	RR (95% CI)	p-value	RR (95% CI)	p-value
Age ≥40 years	6.679 (0.779–57.298)	0.083	NA/NC	
Ephelides	0.110 (0.013‒0.909)	**0.041**	NA/NC	
Previous sunburn	0.444 (0.211‒0.938)	**0.033**	0.626 (0.244–1.610)	0.331
Atypical pigment network/pseudo-network	0.168 (0.068‒0.413)	**<0.001**	0.168 (0.058‒0.491)	**0.001**
Atypical vascular pattern	8.104 (3.488–18.828)	**<0.001**	6.621 (2.462–17.807)	**<0.001**
White shiny lines	13.020 (1.623–104.445)	**0.016**	7.005 (0.682–71.939)	0.101

NA/NC, Not Applicable or Not Calculable; RR, Relative Risk; 95% CI, 95% Confidence Interval.

## Discussion

Of the assessed clinical and epidemiological variables, only the presence of ephelides and a history of previous sunburns were identified as predictive factors for thin melanomas, facts not consistently reported in previous studies.^13^ This result can be explained by the acknowledgment of ephelides and sunburns as risk factors for the development of skin neoplasms and the consequent adoption of secondary prevention measures, such as the use of regular skin self-examination.

Previous studies have shown an association between the atypical pigment network/pseudo-network and thin melanomas, as well as an association between the atypical vascular pattern and white shiny lines with thicker melanomas.^8^, ^9^ However, as opposed to other series, the presence of a blue-gray veil and striae was not associated with melanomas thicker than 1 mm.^8^, ^9^, ^10^

After the multivariate regression, only an atypical vascular pattern and atypical pigment network/pseudo-network were highlighted respectively as independent predicitve factors of melanomas thicker than 1 mm and melanomas with thickness ≤1 mm or less. These associations suggest that more invasive melanomas have greater neovascularization, depicted at the dermoscopic examination as atypical vessels and that melanomas with a thickness ≤1 mm maintain the dermal-epidermal junction intact since the atypical pigment network corresponds to an atypical junctional melanocytic proliferation.^14^

Some study limitations are highlighted, such as the lack of data on some of the assessed variables and the absence of an optimal correlation between the dermoscopic analysis, performed on a horizontal plane, and the anatomopathological examination, performed on the vertical plane. Moreover, in the future, it will be important to carry out prospective cohort studies to confirm the obtained results.

## Conclusions

An incisional biopsy carries the risk of underestimating melanoma thickness, leading to the understaging and eventual inaccuracies in defining the appropriate excision margins and the indication or not of sentinel lymph node biopsy. Dermoscopy is a potentially useful technique for reducing understaging errors, allowing the choice of the most appropriate site for the incisional biopsy, which should avoid areas where there is only an atypical pigment network (related to inferior tumor thickness) and preferably focus on areas where atypical vessels are observed (associated with greater thickness).

## Financial support

None declared.

## Authors' contributions

Ana Rita Silva: Statistical analysis; design and planning of the study; drafting and editing of the manuscript; collection, analysis, and interpretation of data; critical review of the literature.

Ricardo Vieira: Approval of the final version of the manuscript; design and planning of the study; drafting and editing of the manuscript; collection, analysis, and interpretation of data; effective participation in research orientation; critical review of the manuscript; intellectual participation in the propaedeutic and/or therapeutic conduct of the studied cases.

## Conflicts of interest

None declared.
